# Occupational exposure to diesel motor exhaust and risk of lung cancer by histological subtype: a population-based case–control study in Swedish men

**DOI:** 10.1007/s10654-017-0268-5

**Published:** 2017-06-05

**Authors:** Anna Ilar, Nils Plato, Marie Lewné, Göran Pershagen, Per Gustavsson

**Affiliations:** 1 Institute of Environmental Medicine, Karolinska Institutet, Stockholm, Sweden; 2 Centre for Occupational and Environmental Medicine, Stockholm County Council, Stockholm, Sweden

**Keywords:** Lung neoplasms, Carcinoma, squamous cell, Carcinoma, large cell, Diesel exhaust, Occupational exposure, Elemental carbon

## Abstract

We investigated occupational exposure to diesel motor exhaust (DME) and the risk of lung cancer by histological subtype among men, using elemental carbon (EC) as a marker of DME exposure. 993 cases and 2359 controls frequency-matched on age and year of study inclusion were analyzed by unconditional logistic regression in this Swedish case–control study. Work and smoking histories were collected by a questionnaire and telephone interviews. DME was assessed by a job-exposure matrix. We adjusted for age, year of study inclusion, smoking, occupational exposure to asbestos and combustion products (other than motor exhaust), residential exposure to radon and exposure to air pollution from road traffic. The OR for lung cancer for ever vs. never exposure to DME was 1.15 (95% CI 0.94–1.41). The risk was higher for squamous and large cell, anaplastic or mixed cell carcinoma than for alveolar cell cancer, adenocarcinoma and small cell carcinoma. The OR in the highest quartile of exposure duration (≥34 years) vs. never exposed was 1.66 (95% CI 1.08–2.56; *p* for trend over all quartiles: 0.027) for lung cancer overall, 1.73 (95% CI 1.00–3.00; *p*: 0.040) for squamous cell carcinoma and 2.89 (95% CI 1.37–6.11; *p*: 0.005) for the group of undifferentiated, large cell, anaplastic and mixed cell carcinomas. We found no convincing association between exposure intensity and lung cancer risk. Long-term DME exposure was associated with an increased risk of lung cancer, particularly to squamous cell carcinoma and the group of undifferentiated, large cell, anaplastic or mixed carcinomas.

**Electronic supplementary material:**

The online version of this article (doi:10.1007/s10654-017-0268-5) contains supplementary material, which is available to authorized users.

## Introduction

Diesel motor exhaust (DME) is a major source of urban air pollution in Europe [1]. The International Agency for Research on Cancer (IARC) has qualitatively classified DME as carcinogenic to humans, on the basis of epidemiological and experimental studies showing an increased risk of lung cancer in association with DME exposure [2]. The IARC noted that results were not consistent between studies.

Emissions from diesel-fueled vehicles constitute a complex mixture of organic and inorganic compounds, occurring as gases well as particles [3]. The most commonly used indicator substance for DME is nitrogen dioxide (NO_2_) [3, 4]. NO_2_ has the disadvantage that it is also generated from other sources than vehicles and it is not an ideal marker for the carcinogenic properties of DME since it is not carcinogenic in itself [4]. Elemental carbon (EC) has been proposed as a more relevant indicator for DME than NO_2_ [5–8]. Valid exposure–response data for DME are needed as a basis for effective prevention of work-related cancer. A threshold limit value of 20 µg of EC/m^3^ was proposed by the US National Institute of Occupational Safety and Health (NIOSH) in 2003 [9], but the proposal was later withdrawn. No subsequent proposal has occurred even if there is growing evidence for exposure–response in relation to EC [8].

There are few studies of the lung cancer risk associated with specific histological subtypes. The hypothesis that various histologic types of lung cancer might represent different disease entities was first put forward by the Norwegian pathologist Kreyberg, based on observations in nickel refinery workers, where there was a stronger excess of epithelial tumors (squamous cell carcinoma) and small cell carcinomas than of adenocarcinomas and other lung cancer forms [10]. Tobacco smoking is more strongly related to squamous cell carcinoma and small cell carcinoma than to adenocarcinoma [11, 12], and the risk of lung cancer among crocidolite asbestos workers appears more strongly related to suamous cell carcionma than to adenomcarcinoma [13]. Radon exposure in uranium miners seems to be especially related to small cell lung cancer and squamous cell carcinoma [14]. Previous studies on the association between DME and lung cancer showed the strongest associations for squamous cell carcinoma [15–18] and large cell carcinoma [17].

We have earlier reported findings from the population-based Lung Cancer in Stockholm (LUCAS) case–control study, assessing the risk of lung cancer in relation to NO_2_ as a marker of DME [19]. We here report new findings from the LUCAS study, using a recently developed Job-Exposure Matrix (JEM) using EC as a marker of DME. The main objective of this study was to investigate how occupational exposure to DME relates to the risk of lung cancer in terms of exposure–response, using a more specific exposure indicator (EC) than the previously commonly used indicator NO_2_. The second aim was to investigate if occupational exposure to DME was more clearly associated with the histological subtypes earlier reported to be associated with occupational exposures and tobacco smoke, i.e. squamous cell carcinoma and small cell lung cancer than to cancer arising from other cell types.

## Methods

### Study base

This population-based case–control study was conducted within Stockholm County. The study base consisted of men 40–75 years of age living in Stockholm County at any time during 1985–1990. Individuals were not considered part of the study base if they had lived outside of the county for more than 5 years during the time period 1950–1990. Ethical approval for the study was granted by the ethics committee of Karolinska University Hospital.

### Identification of cases and controls

1196 cases and 2765 controls were invited to participate in the LUCAS study. The response rate was 87% among cases and 85% among controls, resulting in 1042 cases and 2364 controls. The methods have been presented in detail earlier [19], but are summarized here.

We identified cases of lung cancer, ICD-7 code 162.1, from January 1, 1985 to December 31, 1990 from the regional cancer register in Stockholm. Information on cancer cell subtypes was obtained from the regional cancer registry. The information was based on notifications to the register from the pathology departments. Information on cell subtype could be obtained for 996 of the 1042 responding lung cancer cases. Among the 996 cases with known histologic cell type, the diagnostic procedure was autopsy with PAD (41.5% of the cases), biopsy (39.3%) or cytology (19.2%). The histologic subtypes were squamous cell carcinoma (n = 398), small cell carcinoma (n = 207), adenocarcinoma (n = 200), undifferentiated, large cell, anaplastic or mixed carcinoma (n = 144), alveolar cell cancer (n = 43), carcinoid (n = 2), leiomyosarcoma (n = 1) and mixed malignant tumour (n = 1).

Controls were identified from a population register. Two groups of controls were selected; population-controls and mortality-matched population controls. Both control groups were frequency-matched with regard to year of inclusion and age in 5-year groups. Mortality-matched controls were additionally matched on vital status as of December 31, 1990. Mortality-matched controls were used to enhance the comparability in the information obtained from cases and controls. These controls were selected among causes of deaths not related to tobacco smoking. We identified next of kin for deceased participants from parish offices or the estate inventories. They were chosen in the following order: spouse, sibling, child, other relative.

### Exposure assessment

We sent a postal survey to participants or next of kin for deceased participants to collect information on work histories and potential confounders. We conducted supplementary telephone interviews with participants who provided incomplete responses to the questionnaire.

Information on age was obtained from the regional cancer register among cases and the population register among controls. Age at inclusion in study was classified into five-years categories (40–44, 45–49…70–75). Smoking was coded as never smoker, former smoker and current smoker (1–10 gm/day, 11–20 gm/day and more than 20 gm/day). We additionally adjusted ever smokers for average grams of tobacco/day among current smokers and average years since quitting smoking among former smokers.

The questionnaire used in LUCAS asked for a life-time occupational history. Occupations were coded according to the Nordic Classification of Occupations [20]. The exposure to DME was assessed by a JEM, specifying the average annual exposure intensity to EC in 72 exposed occupations per calendar year from 1947 to 1994. A more detailed description of the JEM has been published elsewhere [21]. The JEM was applied to the study subjects’ work histories in a case-by-case procedure in order to make full use of the additional information on work tasks and work conditions, e.g. part-time work, that was present in the questionnaires. This information was used to make a more accurate estimation of the exposure probability than using the matrix only. The occupational hygienists doing this were blinded with regard to the participant’s case–control status. We classified cumulative exposure, highest annual average intensity of exposure during at least 1 year of work, duration and years since exposure cessation into quartiles based on the exposure distribution among exposed controls. Cumulative exposure was calculated by taking the product of exposure intensity, probability and years of exposure per work period, summed over all work periods.

Occupational exposure to asbestos and combustion products (other than motor exhaust), as well as air pollution from road traffic and residential exposure to radon, were obtained from the exposure classification of the original study [19, 22]. For occupational cumulative exposure to asbestos, we used a binary variable where the cutoff was set at the median value among the exposed (>0.89 versus 0–0.89 fiber-years/ml). Occupational exposure to combustion products (other than motor exhaust) (μg-years/m^3^ of benzo(*a*)pyrene) was coded in a similar manner as asbestos, where the reference was set at ≤6.69. We used environmental exposure to NO_2_ in order to adjust for air pollution from road traffic, as described by Bellander et al. [23] and previously analyzed by Nyberg et al. [24]. Briefly, air pollution data from a regional emission database combined with dispersion modelling were linked to residential histories. From the annual mean concentration of NO_2_, the average exposure for each individual was coded as low or high exposure, with a cut-off at the 90th percentile. Time-weighted cumulative exposure to residential radon was assessed according to a method described by Pershagen et al. [25] using information on house type and building material from the questionnaire, combined with geographic data on ground radon to predict cumulative indoor radon exposure, dichotomized at the median value.

### Statistical analysis

We used unconditional logistic regression to estimate odds ratios (ORs) and 95% confidence intervals (CIs) of lung cancer for ever vs. never exposure to DME, exposure duration, cumulative exposure, exposure intensity and years since exposure cessation. Tests for trend for each measure of diesel exhaust were performed by assigning the mean value of the exposure metric in each quartile to all persons in that quartile and modeling this as a continuous variable in an unconditional regression model. No systematic differences in findings were observed when comparing the results using the two control groups and both control groups were combined in all analyses.

We evaluated to what extent the risk estimates depended on histological subtype by restricting the cases to alveolar cell cancer, adenocarcinoma, squamous cell carcinoma, small cell carcinoma and the group of undifferentiated, large cell, anaplastic or mixed carcinoma, respectively. This grouping followed the classification scheme used by the Swedish cancer registry. Alveolar cell cancer is only presented in the table of ever exposure; since there were only 6 out of 43 cases that had been exposed to DME. Cases with alveolar cancer, as well as the 4 cases with mixed malignant tumour, carcinoid or leiomyosarcoma were included in the total lung cancer group in all tables. Participants with missing data on confounders (n = 9), were excluded from the analysis, as well as cases with missing data on histological subtype (n = 46). 993 cases and 2359 controls had complete data on histological subtype and/or all confounders and were thereby eligible for analysis.

All analyses were adjusted for age group and year of inclusion in study. We additionally adjusted for potential confounding from tobacco smoking, occupational exposure to asbestos, residential radon, occupational exposure to combustion products (other than motor exhaust) and residential exposure to traffic related air pollution using NO_2_ as marker. In order to test if exposure duration was associated with EC intensity, we further adjusted exposure duration for average annual intensity as a continuous variable. The statistical analyses were conducted with Stata version 14.

## Results

Population characteristics of the 993 cases and 2359 controls are presented in Table 1. As expected, more cases than controls were current smokers, and they smoked more than the controls. Among the 36 never smoking cases, 4 had ever been exposed to DME. A larger proportion of the cases had been exposed to DME, other combustion products than motor exhaust and asbestos at their work, as compared to the controls. For 86% of the 673 DME-exposed participants 20 years or more had passed since first year of exposure and inclusion in the study.

**Table 1:** Baseline characteristics of analyzed 993 cases and 2359 controls in the LUCAS study^a^

Characteristics	Cases n (%)	Controls n (%)	*p* value
Mean age (std. dev.)	66 (7)	66 (7)	*p* = 0.124
Questionnaire answered by			
Participant	68 (7)	1118 (47)	*p* = **0.000**
Next of kin	925 (93)	1241 (53)	
Smoking status (gm/day)			
Never	36 (3)	705 (30)	*p* = **0.000**
Former	273 (26)	844 (36)	
Current 1–10	143 (14)	313 (13)	
Current 11–20	348 (33)	363 (15)	
Current >20	242 (23)	139 (6)	
Average grams of tobacco/day among current smokers	18	14	*p* = **0.000**
Average years since quitting smoking among former smokers	10	18	*p* = **0.000**
Occupational exposure to asbestos			
Never	794 (80)	2020 (86)	*p* = **0.000**
Ever	199 (20)	339 (14)	
Residential exposure to radon			
Low	502 (51)	1172 (50)	*p* = 0.645
High	491 (49)	1187 (50)	
Occupational exposure to diesel motor exhaust			
Never	766 (77)	1929 (82)	*p* = **0.002**
Ever	227 (23)	430 (18)	
Occupational exposure to combustion products (other than motor exhaust)			
Never	784 (79)	1974 (84)	*p* = **0.001**
Ever	209 (21)	385 (16)	
Air pollution from road traffic			
Low	877 (88)	2138 (91)	*p* = **0.042**
High	116 (12)	221 (9)	

Bold values indicate tests for trend were 2-tailed and statistical significance was set at *p* < 0.05

^a^Restricted to participants with data on age group, year of study inclusion, tobacco smoking, occupational exposure to asbestos, occupational exposure to elemental carbon, occupational exposure to combustion products (other than motor exhaust), air pollution from road traffic and residential radon

Table 2 presents the risk of lung cancer among participants ever exposed to DME during their working life. There was an increased unadjusted risk of lung cancer among those ever exposed to DME, but adjustments for the set of potential confounders attenuated the OR (OR 1.15, 95% CI 0.94–1.41). The risk of lung cancer related to DME differed for the various histological subtypes, with significantly increased unadjusted risks for squamous cell carcinoma and the group of undifferentiated, large cell, anaplastic or mixed carcinomas in the crude model. No elevated risks were noted for alveolar cell cancer, adenocarcinoma or small cell carcinoma. When adjusting for the potential confounding factors, the increased risk remained for undifferentiated, large cell, anaplastic or mixed carcinoma (OR 1.57, 95% CI 1.05–2.34) while the OR for squamous cell carcinoma was of borderline significance (OR 1.30, 95% CI 0.99–1.71).

**Table 2:** Ever exposure to diesel motor exhaust and ORs of lung cancer subdivided by lung cancer histologic subtype

	No. of cases/controls	Crude OR (95% CI)^a^	Adjusted OR (95% CI)^b^
All cell types			
Unexposed	766/1929	1.00	1.00
Ever exposed to motor exhaust	227/430	**1.33 (1.11**–**1.59)**	1.15 (0.94–1.41)
Alveolar cell cancer			
Unexposed	37/1929	1.00	1.00
Ever exposed to motor exhaust	6/430	0.74 (0.31–1.77)	0.70 (0.29–1.70)
Adenocarcinoma			
Unexposed	161/1929	1.00	1.00
Ever exposed to motor exhaust	38/430	1.07 (0.74–1.54)	0.99 (0.67–1.46)
Squamous cell carcinoma			
Unexposed	298/1929	1.00	1.00
Ever exposed to motor exhaust	98/430	**1.47 (1.14–1.90)**	1.30 (0.99–1.71)
Small cell carcinoma			
Unexposed	162/1929	1.00	1.00
Ever exposed to motor exhaust	45/430	1.24 (0.87–1.75)	1.06 (0.73–1.55)
Undifferentiated, large cell, anaplastic or mixed carcinoma			
Unexposed	104/1929	1.00	1.00
Ever exposed to motor exhaust	40/430	**1.70 (1.16–2.50)**	**1.57 (1.05–2.34)**

Bold values indicate tests for trend were 2-tailed and statistical significance was set at *p* < 0.05

*CI* confidence interval, *EC* elemental carbon, *OR* odds ratio

^a^Adjusted for age group and year of study inclusion

^b^Adjusted for age group, year of study inclusion, tobacco smoking, occupational exposure to asbestos, residential radon, combustion products (other than motor exhaust) and air pollution from road traffic

The ORs of lung cancer subdivided by number of years exposed to DME during the entire work history are presented in Table 3. The risk of lung cancer increased with increasing number of years exposed to DME in the adjusted model (*p* for trend: 0.027). Among participants exposed to DME for at least 34 years (the highest quartile), the adjusted OR of lung cancer was 1.66 (95% CI 1.08–2.56). The exposure–response relation in the adjusted model remained when restricting the cases to the squamous cell carcinoma cell type (*p* for trend: 0.040). For the group undifferentiated, large cell, anaplastic or mixed carcinoma the OR was 2.89 (95% CI 1.37–6.11, *p* for trend: 0.005) in the highest quartile of exposure duration. The association between number of years of DME exposure and risk of adenocarcinoma was less pronounced with a *p* for trend of 0.093. There was no trend for small cell carcinoma (*p* for trend: 0.508). The adjustment for average yearly DME intensity in the adjusted model had minor effect on the estimates.

**Table 3:** ORs of lung cancer subdivided by number of years exposed to diesel motor exhaust during work

Years with exposure	No. of cases/controls	Average yearly exposure intensity (µg EC/m^3^)	Average number of years exposed	Crude OR (95% CI)^a^	Adjusted OR (95%CI)^b^
All cell types					
Unexposed	766/1929	0	0	1.00	1.00
1–15	50/112	26	8	1.12 (0.79–1.58)	1.02 (0.66–1.58)
16–26	55/105	38	22	1.30 (0.93–1.82)	1.16 (0.72–1.88)
27–33	49/100	37	30	1.18 (0.83–1.68)	1.22 (0.76–1.96)
≥34	73/113	32	37	**1.70 (1.25–2.31)**	**1.66 (1.08–2.56)**
Test for trend	993/2359			*p* = **0.001**	*p* = **0.027**
Adenocarcinoma					
Unexposed	161/1929	0	0	1.00	1.00
1–15	6/112	26	8	0.64 (0.28–1.48)	0.84 (0.31–2.30)
16–26	13/105	38	22	1.51 (0.83–2.76)	1.97 (0.80–4.87)
27–33	5/100	38	30	0.61 (0.24–1.51)	0.97 (0.32–2.92)
≥34	14/113	31	37	1.48 (0.83–2.65)	2.08 (0.91–4.73)
Test for trend	199/2359			*p* = 0.401	*p* = 0.093
Squamous cell carcinoma					
Unexposed	298/1929	0	0	1.00	1.00
1–15	20/112	26	8	1.15 (0.70–1.89)	1.00 (0.55–1.80)
16–26	23/105	40	22	1.39 (0.87–2.22)	1.16 (0.61–2.23)
27–33	24/100	38	30	1.42 (0.89–2.26)	1.39 (0.76–2.54)
≥34	31/113	31	37	**1.96 (1.28–2.98)**	1.73 (1.00–3.00)
Test for trend	396/2359			*p* = **0.001**	*p* = **0.040**
Small cell carcinoma					
Unexposed	162/1929	0	0	1.00	1.00
1–15	12/112	26	8	1.24 (0.67–2.31)	1.03 (0.47–2.23)
16–26	13/105	38	21	1.42 (0.78–2.59)	1.21 (0.51–2.87)
27–33	10/100	38	30	1.11 (0.57–2.18)	1.28 (0.54–3.01)
≥34	10/113	32	37	1.16 (0.59–2.27)	1.23 (0.53–2.87)
Test for trend	207/2359			*p* = 0.351	*p* = 0.508
Undifferentiated, large cell, anaplastic or mixed carcinoma					
Unexposed	104/1929	0	0	1.00	1.00
1–15	8/112	26	8	1.25 (0.59–2.66)	1.15 (0.48–2.76)
16–26	5/105	39	22	0.86 (0.34–2.16)	0.69 (0.23–2.14)
27–33	10/100	38	30	1.84 (0.93–3.65)	1.87 (0.78–4.50)
≥34	17/113	32	37	**2.87 (1.65–4.99)**	**2.89 (1.37–6.11)**
Test for trend	144/2359			*p* **=** **0.000**	*p* = **0.005**

Bold values indicate tests for trend were 2-tailed and statistical significance was set at *p* < 0.05

*CI* confidence interval, *EC* elemental carbon, *OR* odds ratio

^a^Adjusted for age group and year of study inclusion

^b^Adjusted for age group, year of study inclusion, tobacco smoking, occupational exposure to asbestos, residential radon, combustion products (other than motor exhaust), air pollution from road traffic and average yearly intensity

When exploring lung cancer risk in relation to the highest annual average intensity of exposure to DME, we found no evident exposure–response relations, neither for lung cancer overall nor for any of the four investigated histological subtypes (Table 4).

**Table 4:** ORs of lung cancer according to the highest annual average intensity of diesel motor exhaust exposure during at least 1 year of work

µg EC/m^3^	No. of cases/controls	Mean number of years exposed	Mean exposure in class (µg EC/m^3^)	Crude OR (95% CI)^a^	Adjusted OR (95% CI)^b^
All					
Unexposed	766/1929	0	0	1.00	1.00
>0–26	53/108	19	18	1.24 (0.88–1.74)	1.17 (0.80–1.71)
>26–33	55/111	25	29	1.25 (0.89–1.74)	1.08 (0.74–1.56)
>33–47	63/102	27	39	**1.56 (1.13–2.16)**	1.24 (0.86–1.78)
>47	56/109	28	84	1.28 (0.92–1.79)	1.14 (0.78–1.65)
Test for trend	993/2359			*p* = **0.033**	*p* = 0.393
Adenocarcinoma					
Unexposed	161/1929	0	0	1.00	1.00
>0–26	6/108	19	18	0.68 (0.30–1.58)	0.67 (0.28–1.59)
>26–33	15/111	24	29	1.60 (0.91–2.81)	1.44 (0.80–2.60)
>33–47	9/102	27	39	1.06 (0.52–2.13)	0.92 (0.45–1.91)
>47	8/109	28	84	0.90 (0.43–1.87)	0.87 (0.40–1.86)
Test for trend	199/2359			*p* = 0.884	*p* = 0.662
Squamous cell carcinoma					
Unexposed	298/1929	0	0	1.00	1.00
>0–26	24/108	19	18	1.45 (0.91–2.30)	1.44 (0.87–2.38)
>26–33	23/111	25	29	1.33 (0.83–2.13)	1.14 (0.69–1.88)
>33–47	27/102	27	39	**1.76 (1.13–2.74)**	1.42 (0.88–2.29)
>47	24/109	27	85	1.39 (0.87–2.20)	1.24 (0.75–2.04)
Test for trend	396/2359			*p* = 0.059	*p* = 0.252
Small cell carcinoma					
Unexposed	162/1929	0	0	1.00	1.00
>0–26	10/108	19	18	1.10 (0.56–2.14)	1.23 (0.59–2.53)
>26–33	9/111	23	29	0.97 (0.48–1.95)	0.78 (0.37–1.63)
>33–47	13/102	27	39	1.50 (0.82–2.74)	1.03 (0.53–1.99)
>47	13/109	27	85	1.40 (0.77–2.55)	1.30 (0.68–2.49)
Test for trend	207/2359			*p* = 0.170	*p* = 0.484
Undifferentiated, large cell, anaplastic or mixed carcinoma					
Unexposed	104/1929	0	0	1.00	1.00
>0–26	10/108	19	18	1.63 (0.82–3.23)	1.66 (0.81–3.40)
>26–33	7/111	24	29	1.20 (0.54–2.64)	1.04 (0.46–2.35)
>33–47	12/102	27	39	**2.13 (1.13–4.01)**	1.79 (0.92–3.49)
>47	11/109	27	84	1.88 (0.98–3.62)	1.83 (0.92–3.64)
Test for trend	144/2359			*p* = **0.019**	*p* = 0.054

Bold values indicate tests for trend were 2-tailed and statistical significance was set at *p* < 0.05

*CI* confidence interval, *EC* elemental carbon, *OR* odds ratio

^a^Adjusted for age group and year of study inclusion

^b^Adjusted for age group, year of study inclusion, tobacco smoking, occupational exposure to asbestos, residential radon, combustion products (other than motor exhaust) and air pollution from road traffic

The risk of lung cancer in association with cumulative exposure to DME is presented in Table S1. For overall lung cancer, the adjusted OR of lung cancer was 1.49 (95% CI 1.04–2.14) in the highest quartile of cumulative exposure (1021 µg-year/m^3^ of EC). Squamous cell carcinoma was the cell type most strongly associated with cumulative exposure. Table S2 provides the ORs of lung cancer subdivided by years since cessation of exposure to DME. The risk decreased for lung cancer overall, squamous cell carcinoma and the group with undifferentiated, large cell, anaplastic or mixed carcinoma with increasing number of years since the DME exposure stopped.

## Discussion

The main finding in this study was that DME exposure was associated with an increased risk of lung cancer, and more specifically to squamous cell carcinoma and undifferentiated, large cell, anaplastic or mixed carcinoma than to alveolar cell cancer, adenocarcinoma or small cell carcinoma. The risk increased with years of exposure and was stronger among participants who were currently exposed at the time of inclusion in study than among previously DME exposed participants.

Studies on DME exposure and risk of lung cancer have reported inconclusive exposure–response associations in terms of duration and intensity [3]. In this study, the number of years being exposed to DME seemed to be a more important determinant of lung cancer risk than the intensity of the exposure. Our findings implied that 34 or more years of exposure was associated with an increased risk of lung cancer (OR 1.66, 95% CI 1.08–2.56). In a large pooled analysis of case–control studies from Europe and Canada—including the previous results from the LUCAS study [19]-workers considered exposed to low levels of DME during 30 years or more had a smoking-adjusted OR of 1.17 (95% CI 1.07–1.29) for lung cancer [26]. Among workers exposed to higher levels of exposure, the risk was higher (OR 1.45, 95% CI 1.07–1.96). This is similar to our findings, where workers in our fully adjusted model exposed during 27–33 years had an OR of 1.22 (0.76–1.96). With regards to smoking, IARC has suggested that exposure duration is the metric that is most closely associated with lung cancer risk [27].

Considering cumulative exposure, our findings showed an increased risk of lung cancer for the highest quartile with an average exposure of 1781 µg-year EC/m^3^ (OR 1.49, 95% CI 1.04–2.14). Assuming 40 years of working life, this corresponds to an average exposure intensity of 44.5 µg EC/m^3^. When we previously used NO_2_ as a marker of DME in the LUCAS study [19], the highest quartile with an average exposure of 5520 µg-year NO_2_/m^3^ resulted in an OR of 1.68 (95% CI 1.23–2.30) after adjustments for age group, selection year, smoking, residential radon level and exposure to air pollution from road traffic. The development of diesel fuel quality has advanced rapidly in recent years and the risks we have observed in the LUCAS study are based on the conditions that prevailed 20 years ago or more. Three large cohorts - including US transportation, railroad and non-metal mining workers—were the most influential studies for the IARC evaluation [2]. A meta-analysis of exposure–response data from these studies showed that a cumulative dose of 600 µg-year EC/m^3^ was associated with a doubled lung cancer risk [8], thus indicating a somewhat steeper exposure–response curve than in our study.

Squamous cell carcinoma and large cell carcinoma were the subtypes most strongly linked to occupational DME exposure in this study. Both squamous cell carcinoma [15–18] and large cell carcinoma [17] have previously been linked to DME exposure. Like in the present study, DME exposure did not seem to be related to adenocarcinoma of the lung [16, 17] or small cell carcinoma [17]. Smoking has been strongly linked to squamous cell carcinoma and small cell carcinoma, but more weakly to adenocarcinoma [12]. Further research is needed to clarify why smoking but not DME is related to small cell carcinoma. Only 3% of cases and 30% of controls in the LUCAS study were never smokers. Due to lack of power we could therefore not restrict the analyses to never smokers, but one advantage of our study is that we controlled for smoking intensity and years since quitting smoking.

The group of undifferentiated, large cell, anaplastic or mixed carcinoma was associated with an increased risk. Unfortunately, it was not possible to subdivide this subgroup further due to the classification system used by the cancer registry. A disadvantage with this study is that there is no standardized validation made of the histopathological diagnoses. This might cause a non-differential misclassification of the outcome, especially as many pathologists were involved.

Like other methods used to assess historical exposure, JEM´s are known to cause misclassification of exposure. Assessing exposure by a generic JEM for the general population is likely to introduce more misclassification than using an industry-specific JEM [28]. Such misclassification is non-differential between cases and controls, and generally tend to attenuate risks. This may possibly explain why a steeper exposure–response was found in the study based on the three industry-specific occupational cohorts [8] than in the present population-based study. The use of a JEM might also explain why the number of years exposed to DME were more associated to lung cancer risk than the intensity of the exposure, since it is more likely that the exposure intensity level may have been misclassified rather than the reported work history.

The biological mechanism linking DME to lung cancer is most likely multifactorial. Inflammatory and genotoxic effects probably play an important role in initiating mechanisms of carcinogenesis. Genotoxic compounds, like PAHs, can react either directly with DNA, or indirectly by damaging the DNA through the production of reactive oxygen species in the lung tissue [29, 30]. Inhalation of DME has been shown to cause tumor formations [31, 32], and 3-Nitrobenzanthrone, emitted from DME has been shown to induce squamous cell carcinoma in the lungs of rats [31]. Since histological subtypes of lung cancer evolve in different parts of the lung, it follows that not only the genotoxic characteristics of the compounds but also where they deposit, will affect the risks for different subtypes of lung cancer. The causal mechanism linking DME exposure to mainly non-small cancer cell types might be similar to that acting for tobacco smoking, since smoking is strongly related to squamous cell carcinoma [27].

In conclusion, this study showed an association between DME exposure and risk of lung cancer, using EC as a marker of DME. The risk appeared more prominent for squamous cell carcinoma and undifferentiated, large cell, anaplastic or mixed carcinoma than for alveolar cell cancer, small cell carcinoma and adenocarcinoma. Number of years exposed was the metric seemingly most strongly associated with disease risk.

## Electronic supplementary material

Below is the link to the electronic supplementary material.

Supplementary material 1 (PDF 145 kb)
