# Supplementary material for: Occupational exposure to diesel motor exhaust and risk of lung cancer by histological subtype: a population-based case–control study in Swedish men
Source: Eur J Epidemiol. 2017 Jun 5;32(8):711–9. doi: 10.1007/s10654-017-0268-5 (PMC5591361; doi:10.1007/s10654-017-0268-5)
Supplement: Supplementary file 1 — Supplementary material 1 (PDF 145 kb) [file 10654_2017_268_MOESM1_ESM.pdf]

Table S1. ORs of Lung Cancer Subdivided by Cumulative Exposure to Diesel Motor Exhaust During Work.

µg-year/m^3 of EC	No. of cases/controls	Average Number of Years Exposed	Average yearly Exposure intensity (µg EC/m^3)	Average µg-year/m^3 of EC	Crude OR (95 % CI) [a]	Adjusted OR (95 % CI)[b]
**All cell types**						
Unexposed	766/1,929	0	0	0	1.00	1.00
>0-308	52/107	10	21	152	1.23 (0.87-1.73)	1.00 (0.68-1.46)
>308-659	54/108	24	23	489	1.25 (0.89-1.76)	1.12 (0.77-1.63)
>659-1,021	54/108	31	28	839	1.25 (0.89-1.75)	1.03 (0.71-1.50)
>1,021	67/107	32	59	1,781	**1.58 (1.15-2.17)**	**1.49 (1.04-2.14)**
Test for trend	993/2,359				*P* = **0.002**	*P* = **0.036**
**Adenocarcinoma**						
Unexposed	161/1,929	0	0	0	1.00	1.00
>0-308	7/107	9	21	154	0.79 (0.36-1.73)	0.70 (0.31-1.57)
>308-659	12/108	24	23	485	1.33 (0.71-2.47)	1.36 (0.71-2.60)
>659-1,021	5/108	31	28	844	0.55 (0.22-1.37)	0.45 (0.18-1.15)
>1,021	14/107	32	59	1,796	1.61 (0.90-2.88)	1.63 (0.88-3.02)
Test for trend	199/2,359				*P* = 0.248	*P* = 0.354
**Squamous cell carcinoma**						
Unexposed	298/1,929	0	0	0	1.00	1.00
>0-308	20/107	9	21	145	1.21 (0.73-1.98)	0.99 (0.58-1.67)
>308-659	21/108	24	23	485	1.28 (0.79-2.08)	1.21 (0.72-2.04)
>659-1,021	30/108	31	28	838	**1.77 (1.16-2.72)**	1.50 (0.95-2.37)
>1,021	27/107	32	61	1,852	**1.62 (1.04-2.53)**	1.52 (0.94-2.47)
Test for trend	396/2,359				*P* = **0.003**	*P* = **0.026**
**Small cell carcinoma**						
Unexposed	162/1,929	0	0	0	1.00	1.00
>0-308	12/107	10	21	154	1.31 (0.70-2.44)	1.01 (0.52-1.98)
>308-659	14/108	24	23	484	1.53 (0.85-2.74)	1.40 (0.74-2.66)
>659-1,021	7/108	31	29	847	0.77 (0.35-1.69)	0.62 (0.27-1.40)
>1,021	12/107	32	62	1,860	1.33 (0.71-2.48)	1.32 (0.67-2.61)
Test for trend	207/2,359				*P* = 0.411	*P* = 0.638
**Undifferentiated, large cell, anaplastic or mixed carcinoma**						
Unexposed	104/1,929	0	0	0	1.00	1.00
>0-308	9/107	10	20	150	1.54 (0.75-3.14)	1.35 (0.64-2.84)
>308-659	5/108	25	23	481	0.80 (0.32-2.01)	0.77 (0.30-1.99)
>659-1,021	12/108	31	28	848	**2.09 (1.11-3.94)**	1.75 (0.90-3.40)
>1,021	14/107	32	61	1,829	**2.46 (1.36-4.47)**	**2.51 (1.34-4.74)**
Test for trend	144/2,359				*P* = **0.001**	*P* = **0.003**

Abbreviations: CI, confidence interval; EC, elemental carbon; OR, odds ratio.

[a] Adjusted for age group and year of study inclusion.

[b] Adjusted for age group, year of study inclusion, tobacco smoking, occupational exposure to asbestos, residential radon, combustion products (other than motor exhaust) and air pollution from road traffic.

Table S2. ORs of Lung Cancer Subdivided by Years since Exposure Cessation to Diesel Motor Exhaust.

Years since Exposure Cessation	No. of cases / controls	Average Number of Years Exposed	Average yearly Exposure intensity (µg EC/m^3)	Average Number of Years Since Exposure Cessation	Crude OR (95 % CI)[a]	Adjusted OR (95 % CI)[b]
**All cell types**						
Unexposed	766/1,929	0	0	-	1.00	1.00
≥ 19	43/114	10	27	28	0.97 (0.67-1.39)	0.89 (0.60-1.32)
9-18	62/106	24	39	13	**1.44 (1.04-2.00)**	1.07 (0.75-1.54)
1-8	71/134	31	37	5	1.31 (0.97-1.77)	1.21 (0.86-1.69)
Currently exposed	51/76	33	27	0	**1.74 (1.20-2.53)**	**1.60 (1.05-2.45)**
Test for trend	176/354				*P* = 0.145	*P* = 0.230
**Adenocarcinoma**						
Unexposed	161/1,929	0	0	-	1.00	1.00
≥ 19	8/114	10	28	28	0.87 (0.42-1.82)	0.83 (0.39-1.77)
9-18	7/106	24	39	13	0.81 (0.37-1.78)	0.64 (0.29-1.44)
1-8	13/134	30	37	5	1.19 (0.66-2.16)	1.19 (0.64-2.22)
Currently exposed	10/76	34	27	0	1.45 (0.72-2.89)	1.45 (0.70-3.01)
Test for trend	199/2,359				*P* = 0.545	*P* = 0.433
**Squamous cell carcinoma**						
Unexposed	298/1,929	0	0	-	1.00	1.00
≥ 19	15/114	10	28	28	0.86 (0.49-1.50)	0.81 (0.45-1.45)
9-18	27/106	24	40	13	1.53 (0.98-2.39)	1.16 (0.72-1.87)
1-8	29/134	30	37	5	1.32 (0.87-2.02)	1.23 (0.78-1.94)
Currently exposed	27/76	33	27	0	**2.77 (1.72-4.45)**	**2.65 (1.56-4.49)**
Test for trend	71/334				*P* = 0.*149*	*P* = 0.*213*
**Small cell carcinoma**						
Unexposed	162/1,929	0	0	-	1.00	1.00
≥ 19	12/114	10	28	28	1.28 (0.69-2.39)	1.06 (0.54-2.08)
9-18	16/106	24	39	13	**1.80 (1.03-3.13)**	1.30 (0.71-2.35)
1-8	11/134	31	38	5	0.92 (0.49-1.75)	0.88 (0.44-1.75)
Currently exposed	6/76	33	26	0	0.95 (0.40-2.26)	0.97 (0.39-2.43)
Test for trend	207/2,359				*P* = 0.466	*P* = 0.608
**Undifferentiated, large cell, anaplastic or mixed carcinoma**						
Unexposed	104/1,929	0	0	-	1.00	1.00
≥ 19	5/114	10	28	28	0.81 (0.32-2.04)	0.80 (0.31-2.05)
9-18	10/106	24	41	13	1.74 (0.88-3.45)	1.33 (0.66-2.70)
1-8	17/134	31	36	5	**2.35 (1.36-4.06)**	**2.37 (1.33-4.22)**
Currently exposed	8/76	33	26	0	1.84 (0.85-4.00)	1.75 (0.77-3.96)
Test for trend	32/313				*P* = 0.051	*P* = **0.036**

Abbreviations: CI, confidence interval; EC, elemental carbon; OR, odds ratio.
[a] Adjusted for age group and year of study inclusion.
[b] Adjusted for age group, year of study inclusion, tobacco smoking, occupational exposure to asbestos, residential radon, combustion products (other than motor exhaust) and air pollution from road traffic.
